# Money and Mind

**Published:** 1878-11

**Authors:** 


### MONEY AND MIND.

Of five hundred and fifty-one lunatics in Great Britain, there are five hundred and five whose aggregate annual income is near twelve hundred thousand dollars, or about twenty-three hundred dollars each.

In connection with this fact we may state, that of a given number of lunatics in Massachusetts, three-fourths were of parents, one or both of whom drank liquor largely. Extremes meet. The rich, who revel in luxury and ease, and the poor, who riot in rum, furnish the children for the mad-house; thus giving us the strongest reason to infer, that if our race is perpetuated in physical vigor and mental power, it must be done, in the parents, by the practice of temperance and industry: temperance in the indulgence of all the appetites of our nature and industry in the prosecution of our callings

